# Photo-Cured Glycol Chitosan Hydrogel for Ovarian Cancer Drug Delivery

**DOI:** 10.3390/md17010041

**Published:** 2019-01-10

**Authors:** Hoon Hyun, Min Ho Park, Gayoung Jo, So Yeon Kim, Heung Jae Chun, Dae Hyeok Yang

**Affiliations:** 1 Department of Biomedical Sciences, Chonnam National University Medical School, Gwangju 61469, Korea; hhyun@chonnam.ac.kr (H.H.); jky6213@naver.com (G.Y.); 2 Department of Surgery, Chonnam National University Medical School, Gwangju 61469, Korea; mhpark@chonnam.ac.kr; 3 Department of Dental Hygiene, College of Health Sciences, Cheongju University, Cheongju 28503, Korea; goodany@naver.com; 4 Department of Biomedical Sciences, College of Medicine, The Catholic University of Korea, Seoul 06591, Korea; chunhj@catholic.ac.kr; 5 Institute of Cell and Tissue Engineering, College of Medicine, The Catholic University of Korea, Seoul 06591, Korea

**Keywords:** injectable drug delivery depot system, visible light-cured glycol chitosan hydrogel, beta-cyclodextrin, paclitaxel, ovarian cancer therapy

## Abstract

In this study, we prepared an injectable drug delivery depot system based on a visible light-cured glycol chitosan (GC) hydrogel containing paclitaxel (PTX)-complexed beta-cyclodextrin (β-CD) (GC/CD/PTX) for ovarian cancer (OC) therapy using a tumor-bearing mouse model. The hydrogel depot system had a 23.8 Pa of storage modulus at 100 rad/s after visible light irradiation for 10 s. In addition, GC was swollen as a function of time. However, GC had no degradation with the time change. Eventually, the swollen GC matrix affected the releases of PTX and CD/PTX. GC/PTX and GC/CD/PTX exhibited a controlled release of PTX for 7 days. In addition, GC/CD/PTX had a rapid PTX release for 7 days due to improved water solubility of PTX through CD/PTX complex. In vitro cell viability tests showed that GC/CD/PTX had a lower cell viability percentage than the free PTX solution and GC/PTX. Additionally, GC/CD/PTX resulted in a superior antitumor effect against OC. Consequently, we suggest that the GC/CD system might have clinical potential for OC therapy by improving the water solubility of PTX, as PTX is included into the cavity of β-CD.

## 1. Introduction

Paclitaxel (PTX) is one of the most widely used anticancer drugs for epithelial ovarian cancer (OC) chemotherapy [1]. It induces apoptosis in cancer cells by targeting microtubules involved in mitotic spindle formation during cell division, the maintenance of cell structure and motility, and cytoplasmic movement [2]. Over the decades, chemotherapy for OC has been changed to a combination of platinum and taxane drugs from single anticancer drugs via intravenous and intraperitoneal administrations [3]. Despite advances in chemotherapy treatments, there are side effects, including nausea, fatigue, vomiting, and anorexia, as well as the complexity of intraperitoneal administration [3]. For PTX, its limited water solubility causes poor bioavailability. Therefore, water-soluble PTX systems, such as TAXOL^®^ and Genexol^®^ PM Injection, have been clinically applied to treat cancers, but they still result in an unsatisfactory effect because of the use of a toxic excipient (a solvent system of Cremophor^®^ EL and dehydrated ethanol) and insufficient targeting [4].

To overcome these drawbacks, to date, various drug delivery systems and approaches based on polymer- and lipid-based nanomaterials and microspheres, such as poly(lactic acid) (PLA), poly(ε-caprolactone) (PCL), poly(β-benzyl L-aspartate) (PBLA), poly(γ-benzyl L-glutamate (PLBG), and liposomes, for targeting tumors have been developed [4]. These systems have several advantages of passive/active targeting to tumor sites, ease of administration, and prolonged drug release; however, they often cause unsatisfactory results because of rapid clearance, limited half-life, frequent dosing, and limited tumor penetration. Therefore, advanced drug delivery systems have been explored and injectable depot systems are considered a good alternative due to localized and sustained drug release, low systemic toxicity, prolonged stability of drugs, increased bioavailability, and reduced dose dumping [5].

In the present study, to overcome the poor water solubility of PTX and the drawbacks associated with systemic drug delivery systems, we prepared an injectable drug delivery depot system, based on a visible light-cured glycol chitosan (GC) hydrogel containing paclitaxel (PTX)-complexed beta-cyclodextrin (β-CD) (GC/CD/PTX) for the targeted delivery of PTX against OC cells. The polymer, which is a derivative of chitosan extracted from shrimp and crab, can make an injectable, photo-cured hydrogel system by conjugating a functional group that allows for photo-curing to its amine groups [6,7,8]. In addition, β-CD can improve the water solubility of PTX by forming an inclusion complex [9,10]. The antitumor effect of the PTX delivery system for OC therapy was evaluated both in vitro and in vivo.

## 2. Results

### 2.1. Effect of Visible Light Irradiation on the Change in Storage Modulus

**Figure 1:**
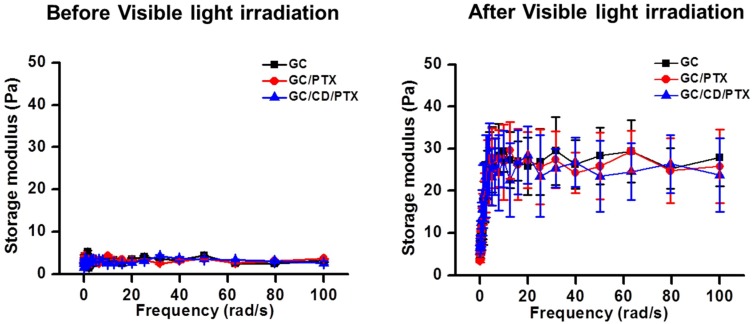
Storage moduli of GC, GC/PTX and GC/CD/PTX (**left** graph) before and (**right** graph) after visible light irradiation for 10 s using blue visible light (430–485 nm). The result was monitored from 0 Hz to 100 Hz. These experiments were carried out for 7 days. The error bars represent mean ± SD (n = 3).

The storage moduli of GC, GC/CD and GC/CD/PTX before and after visible light irradiation were evaluated as a function of frequency using rheometry, as shown in Figure 1. Prior to visible light irradiation, the three samples exhibited between 1 Pa and 5 Pa of storage moduli at all frequencies tested. Irradiation for 10 s to GC, GC/PTX, and GC/CD/PTX resulted in increased storage moduli. At 100 rad/s, GC, GC/PTX, and GC/CD/PTX had storage moduli of 27.9 Pa, 25.8 Pa and 23.8 Pa, respectively.

### 2.2. Swelling Ratio and Degradation Behaviour

**Figure 2:**
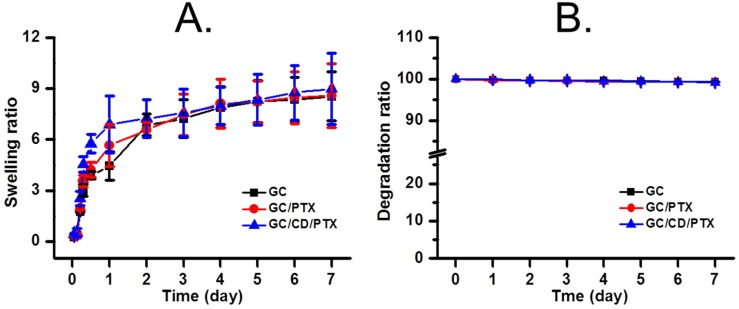
(**A**) Swelling ratios and (**B**) degradation behaviors of GC, GC/PTX and GC/CD/PTX cured using visible light irradiation at 37 °C for 10 s in water. These experiments were carried out for 7 days. The error bars represent mean ± SD (n = 3); * *p* < 0.05.

Figure 2 shows the swelling ratio and degradation behavior of GC, GC/PTX, and GC/CD/PTX cured with visible light irradiation for 10 s in a phosphate-buffered saline (PBS) solution at 37 °C. As shown in Figure 2A, GC, GC/PTX, and GC/CD/PTX exhibited similar swelling behavior during the 7 days tested. The samples were swollen by 5 h and the swollen state was maintained thereafter. The swelling ratios of GC, GC/PTX, and GC/CD/PTX at 5 h of incubation were 8.23-, 8.21- and 8.34-fold higher than those of the initial states, respectively. The degradation behaviors were investigated for 7 days and are shown in Figure 2B. The results showed that GC, GC/PTX and GC/CD/PTX had degradation rates of less than 1% for 7 days, indicating almost no degradation.

### 2.3. In Vitro Release Behavior of PTX

**Figure 3:**
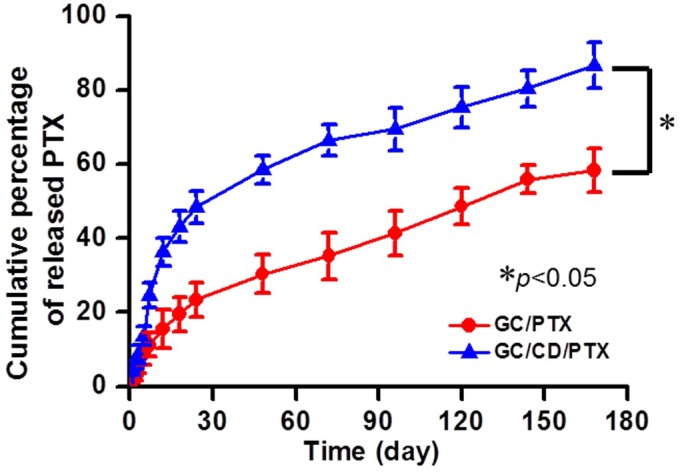
In vitro release behavior of PTX in GC/PTX and GC/CD/PTX at 37 °C for 7 days with continuous shaking at 100 rpm in PBS. The error bars represent mean ± SD (n = 3); * *p* < 0.05.

The release behaviors of PTX in GC/PTX and GC/CD/PTX were investigated at 37 °C for 7 days, as shown in Figure 3. The two samples showed controlled release behaviors in a sustained manner for the periods tested. GC/CD/PTX had a more rapid PTX release than GC/PTX because the solubility of PTX was improved by including the molecule into the cavity of β-CD. The initial bursts were observed within 5 h, and their percentages were 8.5% and 13.6%, respectively. After the initial burst time, PTX was released in a sustained manner due to PTX diffusion from the swollen polymer matrix.

### 2.4. In Vitro Cell Viability

**Figure 4:**
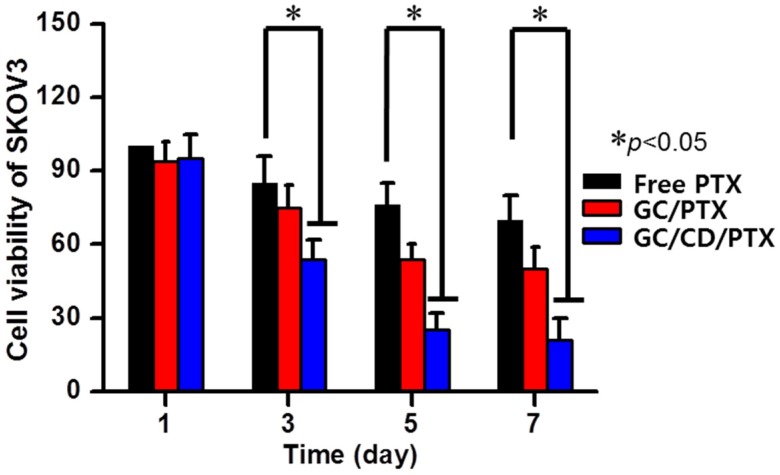
In vitro cell viability of SKOV3 cultured on control, free PTX, GC/PTX and GC/CD/PTX for 1, 3, 5 and 7 days in an incubator set at 37 °C and 5% of CO_2_ using DMEM containing 10% fetal bovine serum, 100 U/mL of penicillin and 100 µg/mL of streptomycin. A specific concentration of cells (5 × 10^3^ cells/well) was first cultured on the samples in 24-well plates. The error bars represent mean ± SD (n = 3); * *p* < 0.05.

The in vitro antitumor effect of GC/CD/PTX was evaluated by measuring the in vitro cell viability percentage of SKOV3 cells cultured on the sample compared with that of the free PTX solution and GC/PTX (Figure 4). Although free PTX, GC/PTX, and GC/CD/PTX decreased cell viability percentages over time, GC/CD/PTX had a lower percentage than free PTX and GC/PTX. At day 1, the cells treated with the samples showed similar viability rates. At day 3, the cell viability rates for GC/CD/PTX were 1.57- and 1.39-fold lower than those for free PTX and GC/PTX. At day 5, GC/CD/PTX had 3.04- and 2.16-fold lower cell viability rates than that of free PTX and GC/PTX. Cells cultured with GC/CD/PTX for 7 days had 3.33- and 2.38-fold lower cell viability rates than those cultured with free PTX and GC/PTX. In addition, compared with day 0, the viabilities of cells cultured with free PTX, GC/PTX, and GC/CD/PTX for 7 days decreased by 70%, 50%, and 21%, respectively. These findings suggest that β-CD improved the water solubility of PTX by the inclusion of complex formation and the photo-cured GC hydrogel has the potential to be a PTX delivery system for OC therapy.

### 2.5. In Vivo Antitumor Effect

**Figure 5:**
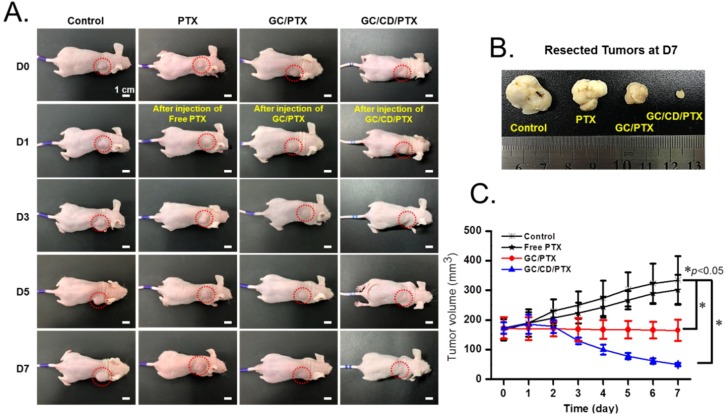
(**A**) Gross appearances of control, and free PTX-, GC/PTX- and GC/CD/PTX-treated tumors observed at days 0, 1, 3, 5 and 7. (**B**) Tumors resected from the animal samples at day 7. (**C**) Tumor volume (mm^3^) of the animal samples measured once a day for 7 days. The tumor volume was calculated by: longest diameter × (shortest diameter)^2^/2. The error bars represent mean ± SD (n = 3); * *p* < 0.05.

Figure 5 shows the in vivo antitumor effect of GC/CD/PTX injected near the tumor in tumor-bearing mice compared with those of free PTX injected intravenously and GC/PTX injected locally. The administrations of free PTX, GC/PTX and GC/CD/PTX were performed once a week. The changes in the gross appearances of the tumor in the control and free PTX-, GC/PTX- and GC/CD/PTX-treated samples are shown in Figure 5A. In the tumors of the control and free PTX-treated samples, the appearances appeared to become larger throughout the tested period. In contrast, local treatments of GC/PTX and GC/CD/PTX led to slight and gradual decrease in tumor size, respectively. To quantitatively investigate tumor growth, tumor volumes at determined time intervals were calculated and are shown in Figure 5B. The tumor volumes in the control and free PTX-treated samples increased gradually, whereas GC/PTX- and GC/CD/PTX-treated samples had a gradual decrease for 7 days. The tumor volumes of the control, and free PTX-, GC/PTX- and GC/CD/PTX-treated samples at day 7 were 2.02- and 1.74-fold higher, and 0.96- and 0.29-fold lower than those at day 0, respectively. Furthermore, the tumor volume of the GC/CD/PTX-treated samples at day 7 was 6.7-, 6.02- and 3.3-fold lower than those of the control, free PTX- and GC/PTX-treated samples, respectively. Figure 5C shows the appearance of tumors extracted from tumor-bearing mice after 7 days. Compared with the control, free PTX- and GC/PTX-treated samples, the tumor size of GC/CD/PTX-treated samples remarkably decreased.

### 2.6. Histological Evaluation

**Figure 6:**
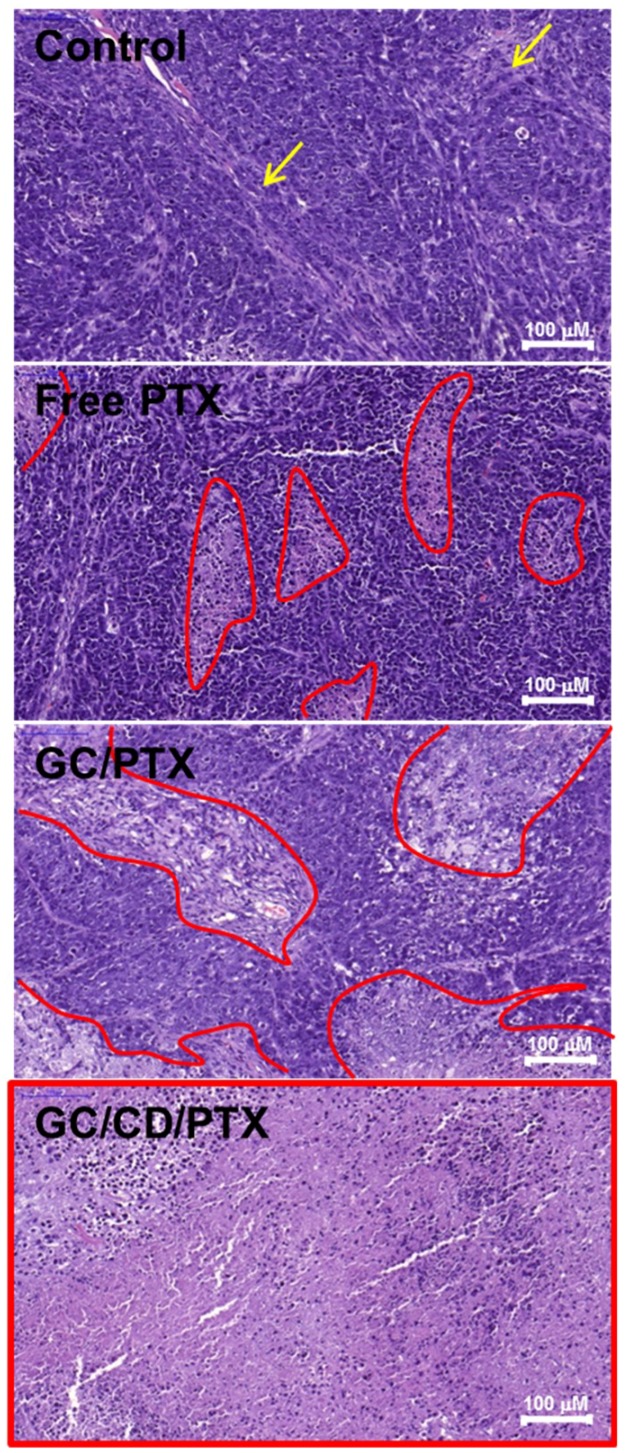
H&E-stained images of control, and free PTX-, GC/PTX- and GC/CD/PTX-treated tumors after 7 days. In control sample, the yellow arrows indicate necrotic tissues induced by limited expansion of tumor tissue in the small animal. In free PTX-, GC/PTX- and GC/CD/PTX-treated samples, the red lines indicate necrotic tissues induced by the apoptosis of cancer cells.

The antitumor effect of the local treatment of GC/CD/PTX was histologically evaluated using hematoxylin and eosin stain and compared with those of the control, free PTX- and GC/PTX-treated samples (Figure 6). The control showed a little necrotic area, which may be attributed to limited expansion of tumor tissue in the small animal. In the hematoxylin& eosin (H&E)-stained image of the control, a lot of viable cancer cells were densely distributed in the tumor tissue. The treatments of free PTX and GC/PTX led to partial necrosis of the tumor tissue. The treatment of GC/CD/PTX noticeably resulted in overall necrosis of tumor tissue. This indicated that the local treatment of GC/CD/PTX improved the antitumor effect against OC by effectively delivering the drug to the targeted site compared to the systemic treatment of free PTX and the local treatment of GC/PTX.

### 2.7. Systemic Toxicity

**Figure 7:**
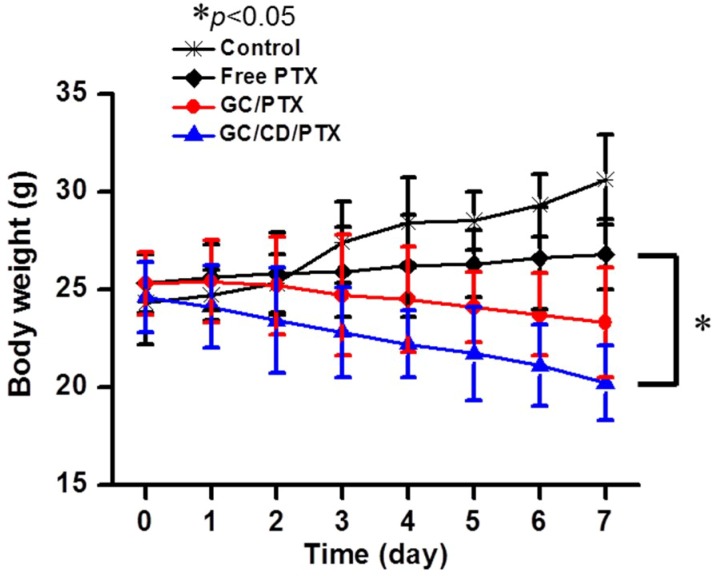
Changes in the body weight of control mice, and mice treated with free PTX, GC/PTX and GC/CD/PTX for 7 days. The body weight was measured once a day. The error bars represent mean ± SD (n = 3); * *p* < 0.05.

The changes in mice body weights in GC/CD/PTX-treated samples were monitored to examine its systemic toxicity and compared with those of the control, free PTX- and GC/PTX-treated samples (Figure 7). Over time, the body weight gradually increased in the control because of tumor growth. In free PTX-treated samples, the body weight slightly decreased. This may not be because of the antitumor effect of PTX, but rather because of the toxicity of the dimethyl sulfoxide (DMSO) that was used to dissolve the PTX. A local administration of GC/CD/PTX resulted in a decrease in body weight because GC can effectively deliver PTX to the targeted site, followed by a decrease in tumor size.

## 3. Discussion

The poor water solubility of PTX generally induces low bioavailability for OC therapy; therefore, strategies for improving the solubility are required. A cyclic compound, β-CD, is known to improve the solubility of PTX by including the drug into the cavity of the ring molecule, because the complex between β-CD and PTX is to be solubilized in water [9,10]. In addition, effective drug delivery systems must be chosen for OC therapy.

Therefore, intratumoral administrations of anticancer drugs have been considered as one of the most effective methods for cancer therapy due to its potential to load and release poorly soluble anticancer drugs; however, it has also caused serious side effects including rapid clearance of applied drugs from tumors and increased dose-limiting toxicity [11,12]. Hence, various drug delivery systems have been developed for parental and topical administrations [13,14,15]. Hydrogels have three-dimensional networks composed of hydrophilic polymeric networks that can contain various anticancer drugs [16].

Among hydrogel drug delivery systems, injectable hydrogels are known to be attractive matrices for the controlled delivery of anticancer drugs to targeted tumor sites. However, thermo-sensitive hydrogels that are widely utilized as injectable systems might cause systemic toxicity because a high dosage of anticancer drugs is released during the initial burst [17]. Because of these problems, in our previous studies, a visible light-cured GC hydrogel system was designed and applied to cancer therapy [6,7,8]. GC has a water solubility because inter- and intramolecular hydrogen bonding was broken by introducing the glycol group to the hydroxyl group of water-insoluble chitosan [18,19]. In addition, functional groups for photo-curing can be conjugated to the amine group of GC through amide bond formation [19]. Additionally, a visible light irradiation system is safer than UV system because visible light does not cause sunburn and malignant melanoma [19]. This visible light-cured GC hydrogel system has been found to have injectability [6].

In this hydrogel system, the initial burst of anticancer drugs was found to be affected by controlling the crosslinking density with the visible light irradiation time [6,7,8]. Thus, a visible light-cured hydrogel system can control the initial burst and has an injectable function [6,7,8]. In the present study, we investigated the potential of visible light-cured GC hydrogel containing PTX for OC therapy.

The controlled release of PTX in GC/CD/PTX in a sustained manner may be attributed to the three-dimensional network of the hydrogel as well as the inclusion of the complex formation between β-CD and PTX. The complexed PTX can be easily released from the polymer hydrogel matrix. Additionally, as explained in our previous studies, visible light-cured GC hydrogel systems have porosity in the surface and bulk of the matrix, which permits the loading of doxorubicin and subsequent drug release in a sustained manner by its diffusion through the swollen matrix in aqueous conditions [6].

As shown in Figure 3, PTX was released from GC/CD/PTX in a biphasic pattern, i.e., initial burst within 5 h followed by a controlled release in a sustained manner. This can be ascribed to the water solubilization of PTX by β-CD and the morphological properties of GC, i.e., the fast and sustained releases can be affected by drugs on or near the matrix surface, and by the diffusion of drugs inside the matrix bulk [20].

To determine whether GC/CD/PTX has antitumor effects in vitro and in vivo, cell viability of SKOV3 cells and tumor volume in the tumor-bearing mouse model were measured. In both experiments, GC/CD/PTX indicated a superior antitumor effect. These results are attributed to some factors as follows: the GC hydrogel injected in the subcutaneous area of nude mouse may be swollen by body fluid and the release of CD/PTX by diffusion. However, in the case of GC/PTX, it had a lower antitumor effect than GC/CD/PTX due to the poor water solubility of PTX during the in vitro experiment and the insufficient targetability of PTX during the in vivo experiment. PTX solubility is known to be approximately 10–20 µM in water; therefore, it limits the cellular uptake into cancer cells, followed by confined cell apoptosis.

**Figure 8:**
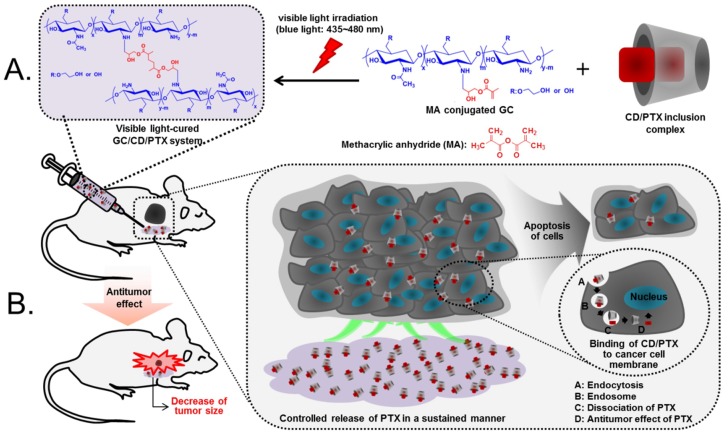
(**A**) Preparation of GC/CD/PTX (**B**) in vivo animal test of the samples using an ovarian cancer-bearing mouse model.

In addition, during the in vivo experiment, PTX molecules in DMSO were irregularly dispersed in normal tissues and tumor sites, because solid tumors have severe barriers to drug delivery due to alterations in the distribution of blood vessels, blood flow, and interstitial pressure [21,22,23]. Eventually, this may cause systemic toxicity due to toxic DMSO and systemic distribution of PTX. Therefore, the visible light-cured GC hydrogel is an outstanding carrier for effective PTX delivery in OC therapy and its local drug delivery system may have potential for clinical use (Figure 8).

## 4. Materials and Methods

### 4.1. Materials

GC (≥60% calculated by titration, crystalline, MW ≅ 585,000 g/mol), glycidyl methacrylate (GM), β-CD, and paclitaxel (PTX) were purchased from Sigma-Aldrich (St. Louis, MO, USA). Riboflavin 5′-monophosphate sodium salt (riboflavin; Santa Cruz Biotechnology, Inc.; Santa Cruz, CA, USA) was used for photo-curing. The cellulose membrane was purchased from Spectrum Laboratories Inc. (Rancho Dominguez, CA, USA). Human OC cell line SKOV3 purchased from the American Type Culture Collection (Manassas, VA, USA) was used for the in vitro cell viability rate and in vivo animal tests. All chemicals were used as received without further purification.

### 4.2. Inclusion Complex Formation between β-CD and PTX (CD/PTX)

To a solution of β-CD (2 mmol, 3 mg) in water (10 mL), PTX (2 mmol, 1 mg) dissolving in acetone (10 mL) was dropped with a continuous agitation for 48 days. After filtration, the transparent solution was lyophilized and stored in a desiccator at −20 °C until subsequent use.

### 4.3. Preparation of Injectable Drug Delivery Depot System

The depot system for OC therapy was prepared using GM-conjugated GC as shown in Figure 8A, which was capable of photo-curing with a riboflavin photoinitiator [6]. Briefly, GC (0.003 mmol, 1.5 g) was dissolved in water and the solution was adjusted to pH 9. To this solution, GM (0.05 mmol, 7 mg) was added and continuously stirred at room temperature for 2 days. After termination, the reactant was neutralized, followed by dialysis (cutoff: 20 kDa) in water for 7 days and lyophilization at −90 °C for an additional 7 days. Riboflavin (12 µM) and CD/PTX (PTX: 2 mg/mL) were homogeneously mixed with GM–GC aqueous solution (1 *w*/*v*%). This mixture was irradiated with blue visible light (430–485 nm, 2100 mW/cm^2^, light-emitting diode curing light, Foshan Keyuan Medical Equipment Co., Ltd., Guangdong, China) for 10 s for hydrogelation (GC/CD/PTX). GC/PTX was prepared by the above-mentioned method.

### 4.4. Storage Modulus Measurement

The storage moduli of hydrogel precursor solutions before and after visible light irradiation for 10 s were measured using a rheometer (AR 2000 EX; TA instruments, New Castle, DE, USA). Prior to irradiation, precursor solutions were placed on a metal mount that was set with a cone and plate geometry with 4 cm of diameter and 1° of cone angle. The moduli were monitored from 0 Hz to 100 Hz.

### 4.5. Measurements of Swell Ratio and Degradation Ratio

GC, GC/PTX and GC/PTX hydrogels prepared in Section 4.3 were immersed in water and extracted at predetermined time intervals. The swelling ratio was calculated as the ratio of the swollen weight at each time interval to the initial weight of the hydrogel. The degradation ratio was expressed as the ratio of the weight of hydrogels at predetermined time intervals to the initial weights.

### 4.6. In Vitro PTX Release Test

GC/PTX and GC/CD/PTX prepared in Section 4.3 were placed in cellulose membrane tubes (cutoff: 3500 g/mol). These hydrogel-loaded tubes were immersed in conical tubes filled with PBS (pH 7.4, 8 mL) and incubated at 37 °C at 100 rpm. At each predetermined time interval (1, 3, 6, 12, and 24 h, and 2, 3, 4, 5, 6, and 7 days), 2 mL of PBS was extracted, and the same volume of fresh PBS was added. The extracted PBS solutions were analyzed using a high-performance liquid chromatographer equipped with a UV detector (1100 series, Agilent Technologies, Palo Alto, CA, USA) and an Ascentis C18 column (25 cm × 4.6 mm, particle size: 5 µm; Supelco, St. Louis, MO, USA). The calibration curve was made with known PTX concentrations. The mobile phase used for this analysis was composed of acetonitrile and water (50:50, *v*/*v*%). The released PTX was detected at 227 nm with a flow rate of 1 mL/min [24].

### 4.7. In Vitro Cell viability Assay

The SKOV3 cell line was used for the in vitro cell viability assay [25,26]. The cells were cultured in an incubator set at 37 °C and 5% of CO_2_ using Dulbecco’s modified Eagle medium containing 10% fetal bovine serum, 100 U/mL of penicillin, and 100 µg/mL of streptomycin reaching 85% confluence. The cells were detached using trypsin and resuspended in the medium at a concentration of 5 × 10^3^ cells/well. After culturing the cell suspension on 24-well plates for 1 h, a free PTX solution (2 mg/mL) in the medium was added. For GC/PTX and GC/CD/PTX, the hydrogels were coated on 24-well plates and the cell suspension was added. The cell viability rates of free PTX, GC/PTX, and GC/CD/PTX were examined using a cell counting kit (CCK-8; 300 µg) reagent at predetermined time intervals (1, 3, 5 and 7 days). After incubation for an additional 2 h, the absorbance of each well was measured using an enzyme-linked immunosorbent assay reader at 450 nm.

### 4.8. Establishment of SKOV3 Xenograft Mouse Model and Evaluation of In Vivo Antitumor Effect

The in vivo animal test is shown in Figure 8B. This animal test was approved by the Institutional Animal Care and Use Committee (IACUC) of Chonnam National University (CNU IACUC-H-2017-64). The animal model was prepared as reported previously [26]. After suspending SKOV3 cells (5 × 10^6^ cells/well) in water for injection (100 µL; JEIL PHARMACEUTICAL Co. Ltd., Daegu, Korea), the suspension was photo-cured for 10s using visible light irradiation and subcutaneously injected into the back of adult male BALB/c nude mice (6 weeks old; n = 6 per group and time interval; 22–25 g; D.Y. Biotech., Seoul, Korea). Mice with a tumor volume of approximately 250–330 mm^3^ were chosen for this experiment. A free PTX solution (2 mg/mL) dissolving in a co-solvent of water for injection (99.9%) and DMSO (0.01%) was intravenously injected through the tail vein. The GC/PTX hydrogel was locally injected near the tumor tissue (approximately 2 mm from the tumor). A specific concentration of PTX (2 mg/kg) was used. The antitumor effect was evaluated by visually and quantitatively analyzing the change in tumor volume at predetermined time intervals. The tumor volumes were calculated using the following formula [27]:

$$\mathrm{Tumor}\;\mathrm{volume}\;\left({\mathrm{mm}}^{3}\right)=\frac{\mathrm{Longest}\;\mathrm{diameter}\times {\left(\mathrm{Shortest}\;\mathrm{diameter}\right)}^{2}}{2}$$

### 4.9. Histological Evaluation

Tumor tissues from tumor-bearing mice after 7 days of treatment were extracted and stained with hematoxylin and eosin (H&E). Prior to staining, the tissues were fixed in 10% of formaldehyde solution overnight, followed by embedding in paraffin. The paraffin-sample blocks were sectioned at a thickness of 4 µm. The slides were visualized by fluorescence microscopy (AX 70, TR-62A02, Olympus, Tokyo, Japan).

### 4.10. Statistical Analysis

All quantitative data are expressed as the mean ± standard deviation. Statistical analyses were performed with one-way analysis of variance (ANOVA) using SPSS software (SPSS Inc., Chicago, IL, USA). A value of *p* < 0.05 was considered statistically significant.

## 5. Conclusions

In the present study, we investigated a new local PTX delivery depot system based on a visible light-cured GC hydrogel cured with riboflavin, which is vitamin B_2_, as a photoinitiator for OC therapy both in vitro and in vivo. This hydrogel system controlled the release of PTX in a sustained manner; therefore, a single local administration of GC/PTX tested for 7 days showed a superior antitumor effect in the OC mouse model than that of systemic administration of free PTX solution via intravenous injection.
